# Stepwise order in protein complex assembly: approaches and emerging themes

**DOI:** 10.1098/rsob.240283

**Published:** 2025-01-15

**Authors:** Michael T. Brown, Michael A. McMurray

**Affiliations:** ^1^ Department of Cell and Developmental Biology, University of Colorado Anschutz Medical Campus School of Medicine, Aurora, CO 80045, USA

**Keywords:** protein complexes, assembly, order, oligomerization, subunits

## Abstract

Protein-based nanomachines drive every cellular process. An explosion of high-resolution structures of multiprotein complexes has improved our understanding of what these machines look like and how they work, but we still know relatively little about how they assemble in living cells. For example, it has only recently been appreciated that many complexes assemble co-translationally, with at least one subunit still undergoing active translation while already interacting with other subunits. One aspect that is particularly understudied is assembly order, the idea that there is a stepwise order to the subunit–subunit associations that underlies the efficient assembly of the quaternary structure. Here, we integrate a review of the methodological approaches commonly used to query assembly order within a discussion of studies of the 20S proteasome core particle, septin protein complexes, and the histone octamer. We highlight shared and distinct properties of these complexes that illustrate general themes applicable to most other multisubunit assemblies.

**Figure:**
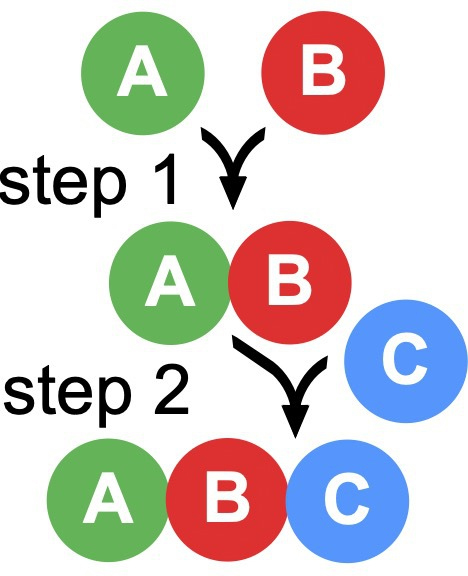

## 1. Introduction

Primary protein structure is readily deduced from DNA/mRNA sequence. With the advent of artificial-intelligence-driven models [1], secondary, tertiary and even quaternary structures can now be predicted with considerable accuracy from sequence alone. What computing is currently unable to accurately predict is the temporal order in which quaternary structure forms; that is, which subunit–subunit interactions happen first, and which happen later. We first discuss reasons why the amino acid sequences of the subunits of a protein complex are insufficient to explain protein complex assembly order.

In the absence of knowledge about the structures of other proteins with similar sequences, for many proteins amino acid sequence alone is insufficient to predict native tertiary structure. Notably, most proteins probably populate a variety of distinct conformations during their lifetimes; a single ‘native conformation’ may be illusory. A major challenge to protein folding *in vivo* is the complex, crowded nature of the cellular environment where most proteins fold. In principle, as it emerges from the ribosome exit tunnel a nascent polypeptide could encounter any of thousands of other molecules. Those encounters may be low affinity, but non-native interactions take a cumulative kinetic toll. Additionally, parts of the polypeptide that in the final native conformation(s) do not touch each other can interact in non-native ways and drive unproductive associations, including irreversible aggregation.

**Figure 1.**
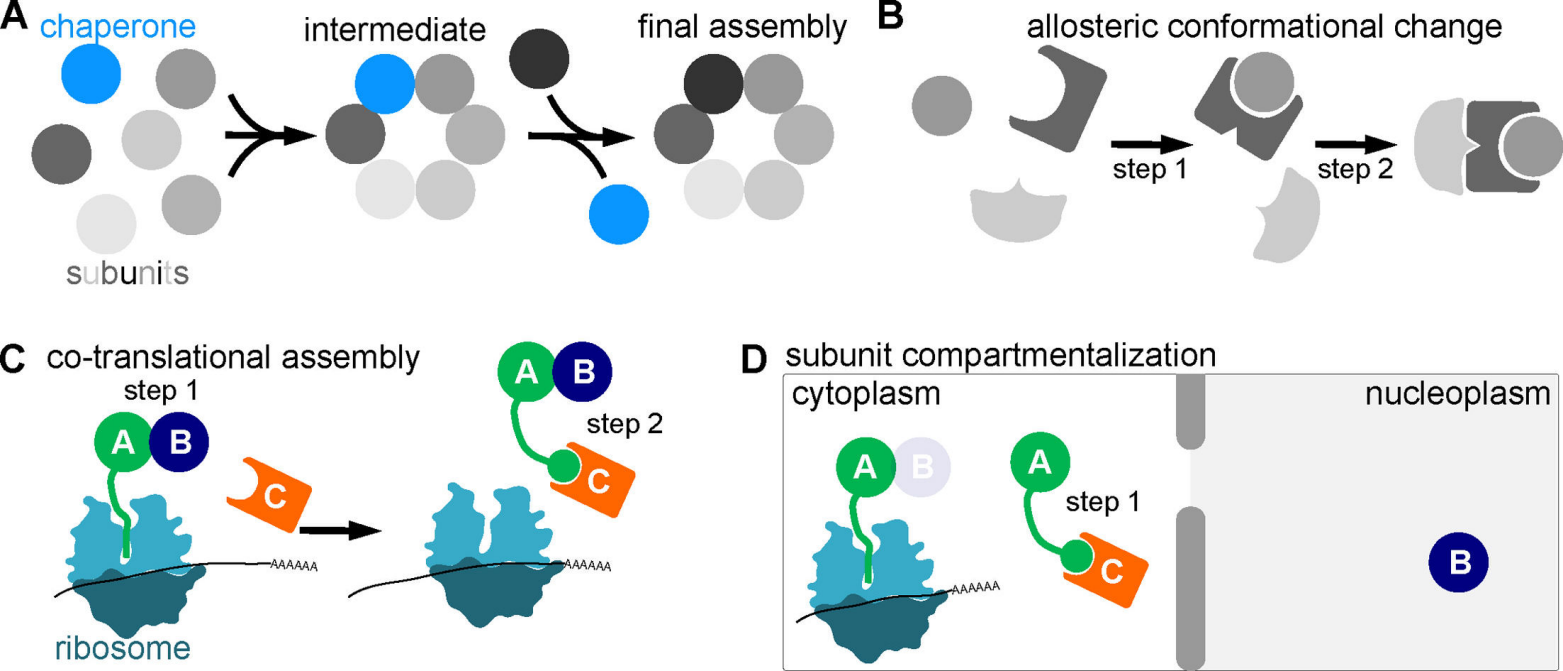
General principles governing assembly order. (A) An assembly chaperone (blue) transiently occupies a subunit–subunit interaction interface. (B) Interaction between two subunits alters the conformation of another interface, allowing a subsequent assembly step. (C) Co-translational interactions between subunits can drive assembly order if distinct assembly interfaces are distantly located in a nascent polypeptide. (D) Subunit compartmentalization, such as primarily nuclear localization of one subunit, can drive assembly order, especially if other steps occur in the cytoplasm during or soon after synthesis.

Cells rely on molecular chaperones to address this challenge. Chaperones are proteins that transiently engage nascent molecules and assist the acquisition of tertiary and/or quaternary structure [2]. Chaperones typically bind to parts of proteins that are initially exposed to solvent but ultimately become buried either within the core of the folded monomer or within an interface between subunits of a stable oligomeric complex. Thus there is an inherent temporal order to chaperone binding: the binding site is available only during a window of time between synthesis of the ‘client’ protein and the point when the client achieves its native tertiary and/or quaternary structure. Here we make a distinction between ‘general chaperones’ and ‘assembly chaperones’. General chaperones are abundant proteins that associate with a diverse range of client proteins at a surface that may or may not eventually become a subunit–subunit interface within a protein complex. Some general chaperones (of the Hsp70, Hsp90 and chaperonin families) actively consume energy by hydrolysing ATP to induce client conformational changes that are unlikely to occur spontaneously. An assembly chaperone is typically dedicated to specific clients and stably occupies a future subunit–subunit interface until the clients have incorporated into their final quaternary structure, thereby promoting a specific assembly order (figure 1A). Assembly chaperones act passively and typically do not consume energy.

One way order can be imposed on an assembly pathway is if the conformation of an assembly interface changes as a result of interaction at a distinct interface. Consequently, one subunit can incorporate into the complex only when other subunits have previously interacted (figure 1B). Allosteric changes (as we refer to them here) are difficult to visualize unless structures of sufficiently high resolution are available for multiple discrete assembly intermediates. Some proteins couple conformational changes to interactions with small molecules, allowing extrinsic regulation. For example, NTPases couple conformational changes to the hydrolysis of nucleotides like ATP or GTP, adopting distinct shapes when bound to ATP/GTP versus ADP/GDP. If interaction between an NTPase and another protein triggers NTPase activity, and the NDP-bound conformation has a high affinity for a third protein, then assembly order readily follows. However, such order cannot currently be predicted from amino acid sequence. When hydrolysis of ATP or GTP is involved, these allosteric changes can be thought of as consuming energy, though these are typically one-time hydrolysis events during assembly, as opposed to repeated cycles of NTP hydrolysis and exchange.

Another way to impose order is if interactions between subunits occur co-translationally, meaning while at least one of the proteins is still undergoing synthesis. Consider subunits A and B, which interact via an interface near the N terminus of subunit A (figure 1C). A also interacts with subunit C via an interface near the C terminus of subunit A. If all the interfaces fold equally quickly, then A and B will interact first. This model assumes that molecules of B and C are available and in oligomerization-competent conformations when the A interfaces fold. If the mRNAs encoding A, B and C are synthesized at around the same time, then this assumption will likely be met if subunit A is the most slowly translated. If subunit A is translated too quickly, then the order imposed by co-translational interactions may be lost. Imposing assembly order via co-translational interactions does not actively consume additional cellular energy.

Finally, two proteins can only interact if they encounter each other. Hence assembly order can also be imposed if specific assembly steps occur in specific compartments or in diffusion-limited regions of a cell (figure 1D). For example, binding of one protein to another may bury a nuclear export signal, allowing the dimer (but not the monomer) to spend long enough in the nucleus to incorporate a third subunit that is always nuclear. Protein trafficking consumes energy, hence imposing assembly order in this way comes at an energetic cost.

Levinthal’s paradox [3] considered how the many theoretically possible folding trajectories faced by a protein seemed to present a problem that would take too long to solve *in vivo*. However, introducing a modest ‘energy cost for locally incorrect bond configurations’ reduces the complexity to something realistically approachable [4]. We think that, once better understood, the properties of proteins interacting in biological systems will similarly allow us to understand how cells reduce the numerous potential assembly pathways to one or a few that optimize fidelity and efficiency. Below we examine three eukaryotic protein complexes that we think illustrate these emerging principles. We use ***bold italics*** to highlight where we specifically address each principle.

## 2. Stepwise assembly of the proteasome core particle

**Figure 2.**
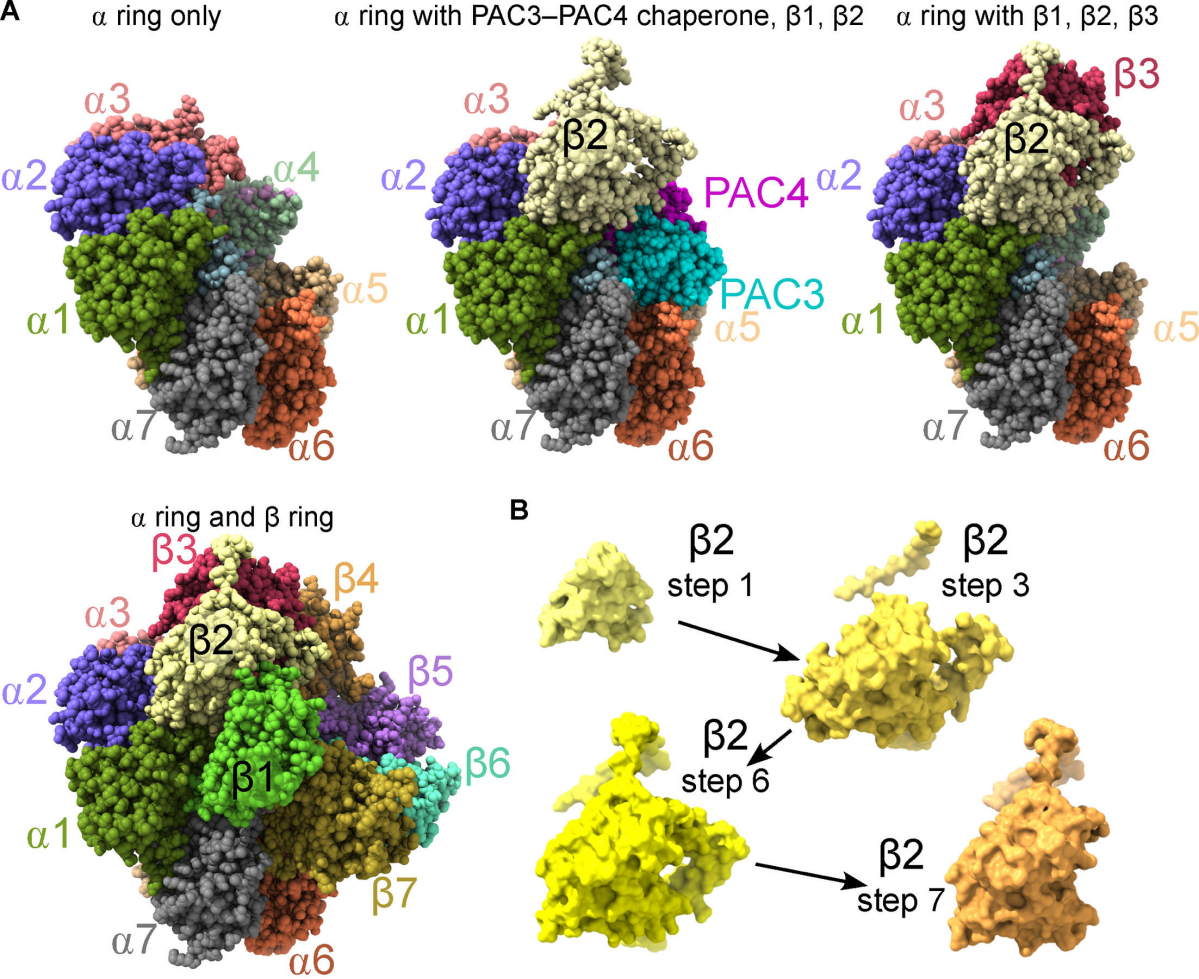
Stepwise pathway of 20S proteasome core particle assembly. (A) A simplified pathway of proteasome core particle assembly showing stepwise addition of a few β subunits onto a pre-assembled ⍺ ring. The complete ⍺ ring is shown at left with associated assembly chaperones hidden. The assembly intermediate in the top centre shows the chaperone PAC3–PAC4 bound mostly to the ⍺5 subunit, plus addition of β1 and β2. At right, addition of β3 is accompanied by eviction of PAC3–PAC4. At bottom, a complete ⍺ and β ring. Protein structures are from PDB 8QYJ, 8QYO and 8QYS and do not necessarily reflect the conformations seen in each solved structure [6]; in some cases, subunits not shown in a complex were present in the solved structures but were simply hidden for this figure. (B) Conformational changes in the β2 subunit during steps along the CP pathway as determined by cryoEM (PDB structures 8QYJ, 8QYM, 8QYS, 8QYO) [6]. ‘Missing’ regions of the protein reflect proteolysis, or poorly structured regions that were not assigned locations.

Proteasomes are among the most abundant protein complexes in cells. They are large, barrel-shaped structures with regulatory caps that dictate which proteins enter the barrel for proteolysis in the central core [5]. Proteasomes represent the main protein maintenance machinery of the cell. The core particle (CP) of the proteasome is composed of stacked heptameric rings of ⍺ and β subunits (figure 2A). CP assembly has been reviewed elsewhere [7,8]. Here we highlight features relevant to assembly order. Whereas in archaea the rings are homoheptamers, in eukaryotes 14 distinct genes encode the 14 CP subunits. Archaeal CP subunits expressed in *E. coli* can self-assemble without the aid of chaperones [9]. The diversification of subunits during evolution presumably presented a challenge to the fidelity of CP assembly, with one ‘correct’ way to assemble but a multitude of possible ‘incorrect’ assemblies involving similar subunit–subunit interfaces. Consequently, eukaryotic CPs do not efficiently self-assemble and require the assistance of multiple ***assembly chaperones*** [7]. Proteasome assembly *in vivo* is apparently somewhat slow, because sub-complexes that are presumably assembly intermediates (and not dead-end/off-pathway or disassembly products) are detectable in wild-type cells via glycerol gradients [10], size exclusion chromatography (SEC) or native PAGE [11] (see Box 1). The ⍺ ring assembles first and β subunits incorporate later; β ring assembly is templated by the ⍺ ring [7,8] (figure 2A). Fast ⍺ ring assembly in the cytoplasm means that ⍺ ring intermediates are hard to detect. Without a way to trap such intermediates or engineer them to generate a specific signal, the assembly order of the ⍺ ring remains unknown.

Box 1. **Experimental approaches used to determine assembly order.**

Several ‘wet lab’ experimental approaches are well suited to investigate protein complex assembly order. Here we illustrate methods for analysis of a hypothetical trimer of protein subunits A, B and C. (See Acknowledgements for graphics credits.) *Electrospray mass spectrometry* of native proteins is able to determine the composition and oligomeric state of protein complexes in a solution [12]. If the protein complex is destabilized prior to analysis, then electrospray mass spectrometry accurately identifies the disassembly products; if disassembly follows the reverse pathway from assembly, then assembly order can be determined [13]. *Cryo-electron microscopy* directly observes protein complexes and if a complex mixture of assembly and/or disassembly intermediates is analysed, an assembly pathway can be inferred and visualized at high resolution. *Native polyacrylamide gel electrophoresis* (native PAGE), *size exclusion chromatography* (SEC, also called gel filtration) and *density gradient sedimentation* (typically glycerol or sucrose) all separate proteins and protein complexes by size and, to some extent, shape. As illustrated here, if protein subcomplex A–B is readily detected but not subcomplex B–C, then it is likely that the A–B interaction normally occurs first, followed by addition of subunit C to create the trimer. Finally, chronological substrate depletion bimolecular fluorescence complementation (CSD-BiFC) is currently the only method capable of determining assembly order in living cells. CSD-BiFC uses split fluorescent proteins and exploits the irreversibility of reconstitution (‘complementation’) and the ability of a common fragment to combine with either of two alternative fragments to create fluorescent proteins of different colours [14]. Cells co-expressing three subunits fused to appropriate fragments are imaged to quantify fluorescent signals, and these signals are compared to signals from cells co-expressing only two tagged subunits. The comparison reveals which subunit–subunit interaction happened first and thereby depleted the potential fluorescence signal from the subsequent subunit–subunit interaction. Note that CSD-BiFC is designed for subunits of 1:1:1 stoichiometry within a complex and may not work for other subunit stoichiometries.
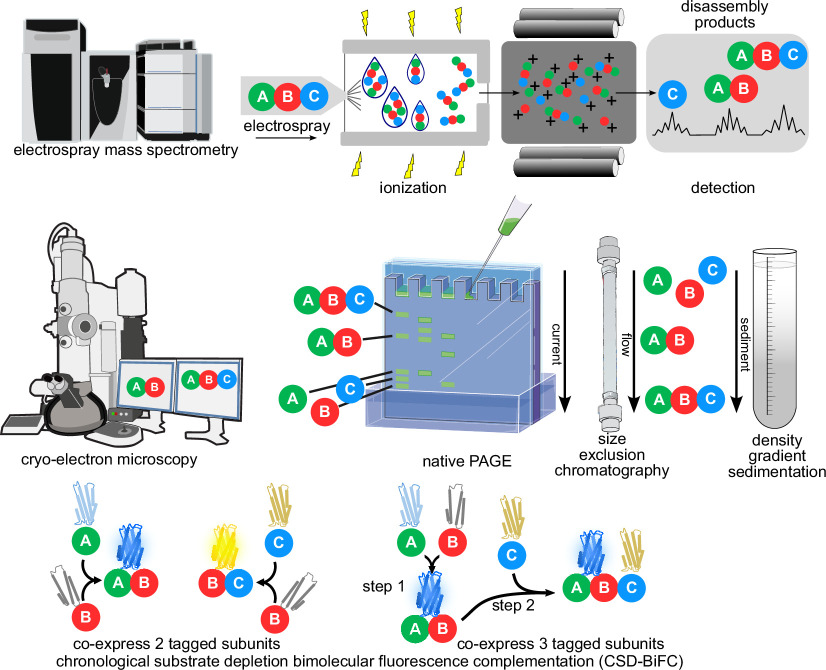

Evidence for step-wise assembly of the β ring, on the other hand, came from studies of intermediates that accumulate when specific β subunits are depleted [15]. In the earliest intermediate, β2 is present on the ⍺ ring, then larger intermediates accumulate β subunits in the order of their naming, except for β1, which arrives with β7 [6]. Several proteasome subunits cleave themselves during assembly, with the extra N-terminal sequences present on the propeptides promoting proper assembly. Thus, these propeptide-specific sequences function as ‘intramolecular chaperones’ [16], because they promote, but are ultimately not constituents of, the native assembly. More traditional ***assembly chaperones*** also participate in CP assembly. Of particular note is the PAC3–PAC4 (Pba3–Pba4 in yeast) heterodimer, which binds to ⍺5 until displaced by the β3 subunit [6,8] (figure 2A).

The β ring assembly order had been worked out but until recently the mechanistic basis was unclear. A cryoEM study visualized eight human CP complexes co-expressed in and purified from insect cells then separated via SEC [6]. The resolution of these structures allowed the identification of conformational changes and contacts between distant subunits mediated by propeptide sequences. Figure 2B shows conformational changes by the β2 subunit during assembly. As the authors note, ‘each incoming subunit ‘structures’ or rigidifies its neighbouring subunit or chaperone in a templating-like manner’, and ‘insertion of each new subunit not only stabilizes the existing assembly intermediate, but also promotes the formation of the next intermediate until the β-ring is complete’ [6]. Notably, even though the major contacts between PAC3–PAC4 and the ⍺ ring are with ⍺5 (figure 2A), PAC3–PAC4 is evicted when the β3 subunit arrives because part of the β2 subunit that contacts PAC3–PAC4 overlaps with a contact between β2 and β3 [6]. The general principles illustrated in assembly of the β ring of the proteasome CP—***assembly chaperones*** that transiently occupy subunit–subunit interaction interfaces and ***allosteric conformational changes*** occurring upon subunit–subunit interactions—are undoubtedly at play in the stepwise assembly of other multisubunit assemblies. It is easy to imagine how a mutation in one β subunit could have no effect on the interface(s) by which it incorporates into the complex, but could prevent the conformational changes required for incorporation of another subunit. Thus understanding the molecular basis of assembly order is crucial to understanding the molecular basis of mutations that cause disease by perturbing complex assembly. (We note that another very recent cryoEM study [17], published while this review was in preparation, provides independent support for the β ring assembly steps and also reveals mechanistic insights about the roles of other chaperones.)

Importantly, the cryoEM approach to resolve human β ring assembly worked because those are the slow steps; ⍺ ring assembly is too fast/efficient. In budding yeast cells, ⍺ ring assembly is slow enough to allow the detection of subcomplexes by SEC of lysates, and here general ***cytosolic chaperones*** were found [18]. These chaperones were required for the detection by native PAGE of complexes containing single ⍺ subunits [18]. Those authors proposed that the general chaperones bind weakly to ⍺–⍺ interfaces. Many ⍺ subunits retain a latent ability to homo-oligomerize. General chaperones may increase the efficiency of native ⍺ ring assembly because ‘improper interactions are likely to be more unstable than correct ones initially, allowing chaperones to engage again after dissociation’ [18]. Analogous to how the native ⍺–β interactions effectively ‘outcompete’ the interactions between ⍺5 and the PAC3–PAC4/Pba3–Pba4 assembly chaperone, native ⍺–⍺ interactions likely outcompete non-native interactions between ⍺ subunits and general chaperones. In this model, general chaperones maintain ⍺ subunits in states competent for native assembly.

Interestingly, the latent capacity for ⍺ homodimerization also has a native context. Yeast studies revealed that the ⍺3 subunit, which is normally sandwiched between ⍺2 and ⍺4 (figure 2A), can be replaced by an extra ⍺4 molecule, creating an ⍺2–⍺4–⍺4 sandwich that alters proteasome function [19]. Such ‘alternative’ proteasomes assemble not only in yeast cells lacking the ⍺3 gene, but also in cells lacking Pba3–Pba4, which normally promotes ⍺3 incorporation [20]. The chaperone-mutant yeast cells are resistant to certain stresses (metal ions) [20], suggesting that the ‘alternative’ assembly may sometimes be made in wild-type cells and has a particular function. Indeed, the analogous alternative assembly is made in human cells and is important for metal ion stress resistance [21]. This example illustrates how plasticity in multisubunit complex assembly can be beneficial, a point further made in the case of septin protein complexes, as discussed below.

While there is (yet) no evidence of co-translational CP assembly, two subunits of the regulatory particle of the proteasome are known to undergo highly choreographed ***co-translational assembly*** [22]. Rpt1 and Rpt2 directly interact in the native proteasome but when synthesized individually in *E. coli*, each is insoluble; co-expression or mixing lysates increases the solubility and allows binding [23]. In yeast and human cells, the mRNAs encoding the Rpt1 and Rpt2 subunits co-localize, suggesting that the two subunits are normally translated in proximity to each other [22]. Translation pause sites ensure that interaction domains in the N terminus of each protein are able to associate before the proteins are fully synthesized and released from the ribosome. Those authors speculated that ‘this mechanism might be useful in particular for proteins that do not readily associate when produced separately’ [22]. Co-translational interactions directly drive assembly order in this scenario.

## 3. Stepwise assembly of linear septin octamers

**Figure 3.**
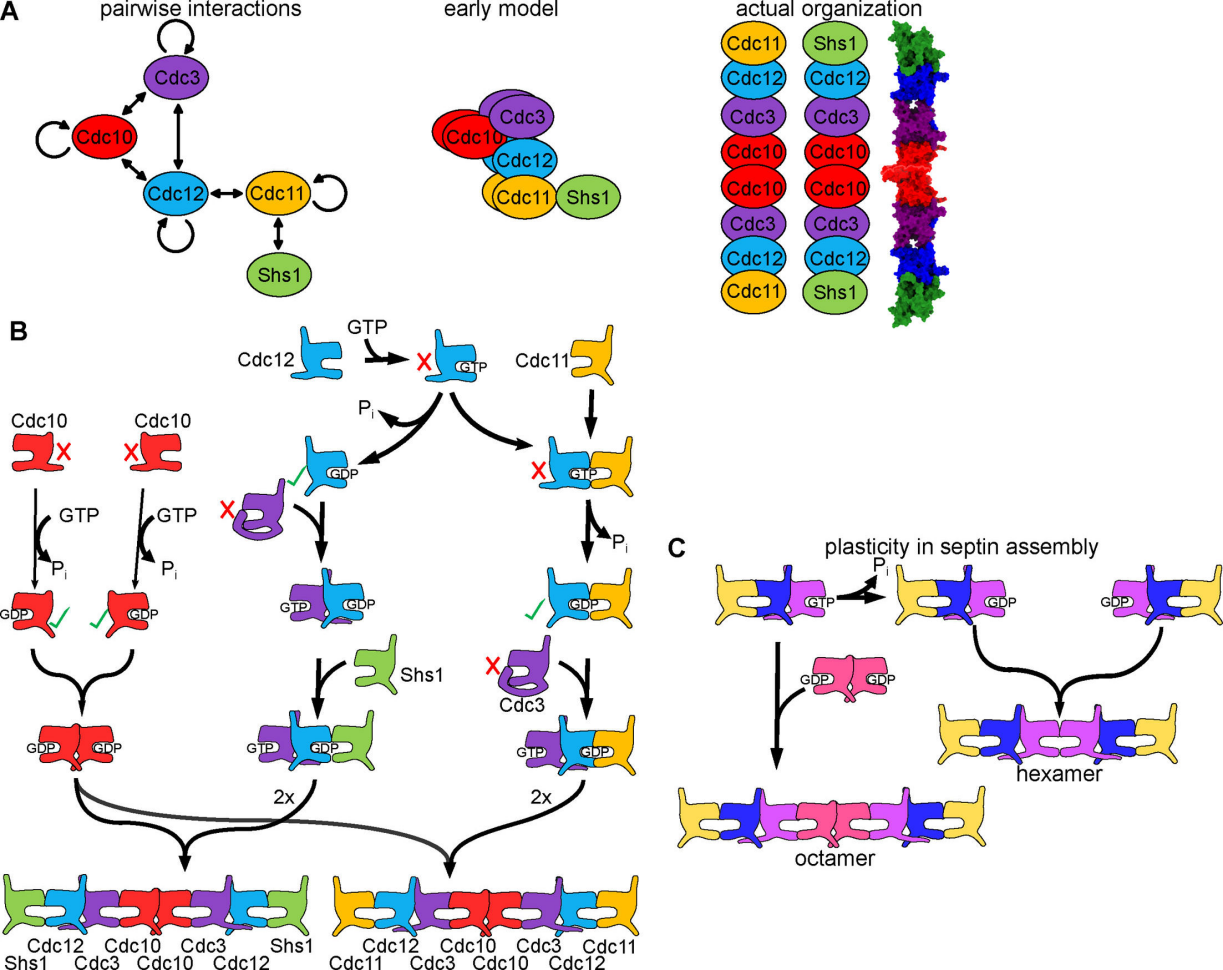
Stepwise pathways for septin complex assembly. (A) *Left*, illustration of pairwise interactions between purified individual budding yeast septins [27], with curved arrows indicating homotypic interactions. *Middle*, the early model for budding yeast septin organization within hetero-oligomeric complexes. *Right*, the actual organization of septin hetero-oligomers, including a representation of the structure of an actual hetero-octamer based on PDB 8PFH [28]. (B) Stepwise assembly pathways of budding yeast septin hetero-octamers, as determined by CSD-BiFC [14]. Cartoons represent septin subunits as in (A). The red ‘X’ and green check marks illustrate allosteric conformational changes that ‘activate’ a specific interaction interface. (C) Two alternative septin assembly pathways are active in organisms in which the purple subunit is able to hydrolyse GTP to GDP, driven by distinct affinities of the GTP- versus GDP-bound conformations of the purple subunit for (*left*) the pink subunit versus (*right*) another GDP-bound molecule of the purple subunit [29]. Portions of this figure were adapted from [14] under license CC BY 4.0.

Our lab has focused prior work on understanding protein complex assembly using the septin family of eukaryotic proteins. Septins co-assemble into hetero-oligomers that further polymerize into cytoskeletal filaments often associated with cellular membranes, where they regulate membrane dynamics [24]. One reason septins are a compelling model comes from the history of how the organization of subunits within budding yeast septin complexes was determined. Five budding yeast septin proteins—Cdc3, Cdc10, Cdc11, Cdc12 and Shs1—were known to co-localize in cells and to co-purify in roughly stoichiometric complexes [25,26] and the purified complexes had been shown to polymerize *in vitro* into filaments resembling the filaments found in yeast cells at the site of septin localization [26]. Thus the purified complex(es) of the five septins was sufficient to recapitulate the presumed higher order structural properties of the endogenous complex(es). A systematic approach by the lab of Jeremy Thorner involved expressing affinity-tagged versions of all five septins individually in *E. coli* [27], which lacks endogenous septins. The purified septins were assumed to be monomeric and were then mixed pairwise with the other purified septins fused to distinct affinity tags. Subsequent co-purification was taken as evidence of direct septin–septin interaction [27]. Every septin showed evidence of weak homotypic interaction. Additionally, Cdc3 bound strongly to Cdc10 and Cdc12; Cdc10 bound strongly to Cdc3 and Cdc12; Cdc11 bound strongly only to Cdc12; and Shs1 bound strongly only to Cdc11 [27] (figure 3A). The only model compatible with these pairwise interactions predicted a short, wide, nine-subunit complex containing homodimers of all septins except Shs1, with Cdc10 simultaneously interacting with Cdc3, Cdc12 and another molecule of Cdc10 (figure 3A) [27].

Only when the Thorner and Nogales labs co-expressed all four yeast septins in the same *E. coli* cells was the true subunit organization revealed: two species of linear hetero-octamers, Cdc11–Cdc12–Cdc3–Cdc10–Cdc10–Cdc3–Cdc12–Cdc11 and Shs1–Cdc12–Cdc3–Cdc10–Cdc10–Cdc3–Cdc12–Shs1 [30] (figure 3A). The lower stoichiometry of Shs1 in complexes purified from yeast simply reflects a lesser abundance of Shs1-containing octamers *in vivo*. The pairwise interaction experiments had been misleading because they imposed an assembly order: each individual septin was synthesized in the absence of other septins and then introduced to only one other septin at a time. Since all septins evolved from a common ancestor that was likely a homodimer, all septins presumably retain the ability to homodimerize if the native septin partner is unavailable, and in some cases two purified septins strongly interacted (e.g. Cdc10 and Cdc12) even if in the native context they do not touch. Notably, by including some co-expression experiments, the early study found hints of the native interaction order: Cdc10 interacted much better with Cdc3 if Cdc12 was also present during expression/assembly [27]. Whereas the authors understandably interpreted this result as strong Cdc10 interactions with both Cdc3 and Cdc12, later experiments directly testing assembly order (see below) provided evidence that Cdc12 binding to Cdc3 induces allosteric changes that expose the Cdc3–Cdc10 interface [14]. Thus, particularly when the subunits share a common ancestor, reliance solely on *in vitro* interactions between individual subunits in the absence of others runs the risk of inspiring faulty models.

*E. coli*-expressed septin complexes have apparently native structures, suggesting no assembly chaperone is required for septin assembly. Reminiscent of the proteasome CP ⍺ ring, septin octamers assemble quickly, such that subcomplexes are scarce in wild-type cells. Septins were first discovered via genetic screens for temperature-sensitive yeast mutants [31]. Our lab found that almost all such septin mutants harbour single substitutions in one of the two assembly interfaces: the ‘G’ interface, which for most septins includes a GTP-binding pocket [32]. We and others discovered by a combination of genetic interactions, SEC, *in vivo* photo-crosslinking [33,34], and ribosome profiling [35] that general cytosolic chaperones specifically bind the nascent septin G interface, acting much like what was proposed for proteasome CP ⍺ subunits. Thus the first step in septin octamer assembly is engagement of the G interface by ***general chaperones***, which likely help prevent the non-native, off-pathway homotypic interactions that were seen with purified septins.

We were interested in directly testing septin assembly order *in vivo* and devised a novel assay for this purpose. GFP and its derivatives of other colours can be split into two fragments that are non-fluorescent but, if fused to other proteins that directly interact, can irreversibly fold together to reconstitute a fluorescent protein [36]. This approach, called BiFC (bimolecular fluorescence complementation), had been expanded by introducing a small number of mutations in the larger of the two fragments to change the colour of the reconstituted protein from green to yellow or blue [37]. We realized that we could determine assembly order by co-expressing three tagged proteins; the protein pair that interact first will dictate the colour of the resulting fluorescence by ‘depleting’ the availability of the small neutral fragment for participation in subsequent interactions (see Box 1). We named the assay CSD-BiFC (for chronological substrate depletion-BiFC) and used it to determine the stepwise assembly order of otherwise wild-type subunits of a protein complex in living cells [14].

The septin octamer assembly pathway offered a few surprises. Based on the previous literature, we expected the G interface to always associate first, followed by the interface on the opposite face of the globular septin GTPase domain, called the NC interface. This expectation derived from indirect evidence in the literature that, for human septins, GTP hydrolysis triggers an ***allosteric conformational change*** in a key component of the NC interface, ‘priming’ the NC interface for interaction [38,39]. However, our CSD-BiFC data showed that NC priming by prior G dimerization could only explain one step: Cdc10 homodimerization in the centre of the octamer (figure 3B). Intriguingly, our observation that an NC interface between Cdc3 and Cdc12 occurred prior to G-mediated interaction between Cdc3 and Cdc10 provided the new assembly order perspective to interpret the interaction data that was seen previously and viewed as evidence of direct binding of Cdc10 to both Cdc3 and Cdc12 (see above). By deleting an N-terminal extension that is unique to Cdc3, we randomized the Cdc12–Cdc3–Cdc10 assembly order, which we interpreted to mean that the N-terminal extension of Cdc3 occludes the Cdc3 G interface and prevents Cdc10 binding there until Cdc12 binds Cdc3 across the NC interface, repositioning that part of Cdc3 and allowing Cdc10 to bind [14]. This septin assembly step is reminiscent of the ***allosteric changes*** that drive order in proteasome CP β ring assembly.

The second surprise presumptive allosteric changes directly related to GTP hydrolysis. Although they are paralogs and compete for the same ‘terminal’ subunit positions in septin octamers (figure 3A), Cdc11 and Shs1 assemble in different orders. Cdc11 interacts with Cdc12 prior to Cdc12–Cdc3 interaction, whereas Shs1 interacts after Cdc12–Cdc3 interaction (figure 3B) [14]. Based on effects of mutations that influence GTP hydrolysis or the accompanying ***conformational changes*** at the G interface, we concluded that intrinsically slow GTPase activity by nascent Cdc12 drives the difference in order: Cdc11 has highest affinity for Cdc12 that has bound GTP but not hydrolysed it, whereas Shs1 has highest affinity for Cdc12 bound to GDP [14]. A similar ‘timer’ mechanism has been previously proposed for the function of other slow GTPases, such as Rab5 regulation of the kinetics of membrane docking and fusion [40]. Furthermore, the GTPase mechanism we propose for septin assembly order is conceptually similar to kinetic proofreading during translation, wherein slow GTP hydrolysis by EF-Tu adds some time between initial aminoacyl-tRNA binding and locking of the tRNA in the decoding centre [41]. This time period allows near- or non-cognate tRNAs to dissociate, improving translational fidelity. The distinction that drives assembly order (versus fidelity) in the septin case is that the pre- and post-hydrolysis interaction interfaces are both ‘productive’ for subunit incorporation into the final complex. Finally, by coupling septin assembly to the cytoplasmic GTP:GDP ratio, cells are able to alter the ratio of Shs1-capped to Cdc11-capped octamers in response to changes in cellular metabolism [14]. Thus in certain cases assembly order is subject to extrinsic regulation.

Septin assembly is also plastic in ways similar to the proteasome CP ⍺ ring. In addition to octamers, many organisms (including humans) make septin hetero-hexamers lacking the central homodimer [42,43]. We found evidence that hexamer assembly involves a distinct conformation of the G interface corresponding to Cdc3–Cdc10 and involving homodimerization of the subunit in the Cdc3 position, bypassing incorporation of the Cdc10 homolog [29] (figure 3C). Because Cdc3 is GTPase-dead and Cdc3•GTP homodimerizes poorly, budding yeast cells make only octamers, but in other organisms a slow GTPase in the Cdc3 position likely drives assembly of a mix of hexamers and octamers (figure 3C), also potentially under metabolic control via the GTP:GDP ratio [29]. Assembly order makes sense here: the central (Cdc10) homodimer either brings together two pre-assembled trimers into octamers, or those trimers come together directly, creating hexamers (figure 3*b*,*c*).

We recognized that the irreversibility of BiFC also makes it well suited to detect otherwise transient chaperone–client interactions by ‘trapping’ them *in vivo*. Exploiting this approach, we found that all yeast septins appear to interact with the same suite of ***general chaperones*** [44], which fits with the high structural similarities among septin GTPase domains. Surprisingly, however, the subunit Cdc12 appears to be translated more slowly than the others, potentially due to a higher density of positively charged residues, which may slow exit from the negatively charged ribosome exit tunnel [34]. We proposed that slow translation and a long C-terminal extension make the nascent Cdc12 polypeptide act as a ***co-translational ‘platform’ for chaperone-guided septin octamer assembly***, entirely consistent with the octamer assembly order determined by CSD-BiFC. Post-translational assembly *in vivo* can produce functional octamers, but only if certain general cytosolic chaperones are present [34]. The chaperones may prevent irreversible misfolding or aggregation by full-length septin proteins that missed the opportunity to oligomerize with other septins during Cdc12 translation.

## 4. Stepwise assembly of histone octamers

**Figure 4.**
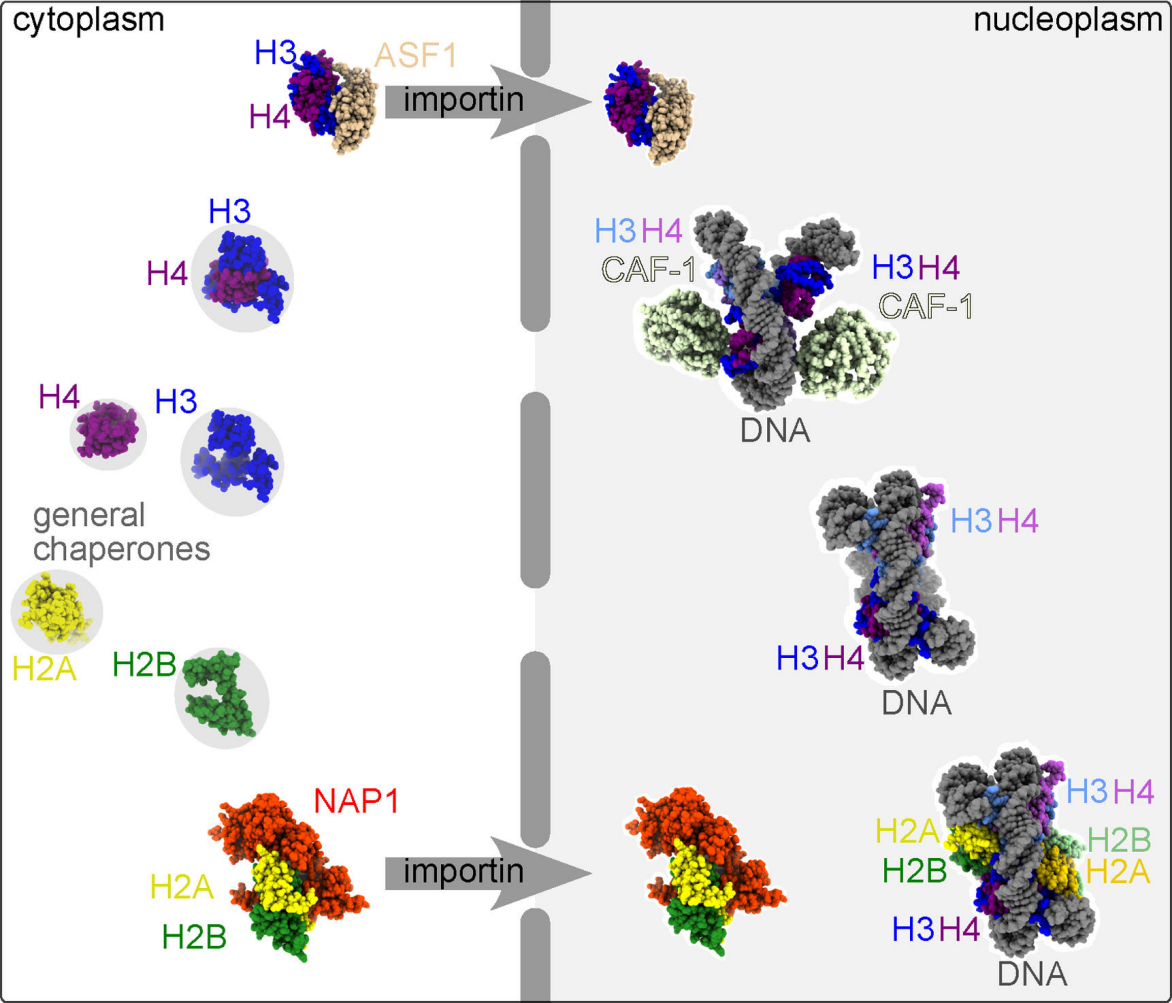
Stepwise pathways for histone octamer/nucleosome assembly. A simplified illustration of the presumptive pathway of histone octamer/nucleosome assembly. Nascent histone monomers are engaged in the cytoplasm by general chaperones (grey circles) before heterodimers are bound by histone-specific assembly chaperones (NAP1 and ASF1) which recruits karyopherins/importins that drive nuclear import. There, H3–H4 dimers are handed off to CAF-1, which brings two heterodimers together with DNA to form a DNA-associated tetramer. NAP1 facilitates incorporation of H2A–H2B dimers into octamers/nucleosomes. Protein structures were based on PDBs 5G2E [45], 1ID3 [46], 8J6T [47] and 2HUE [48].

Histone proteins are small, highly basic proteins. For example, the ‘canonical’ core histones of *S. cerevisiae—*H3 (Hht1/2), H4 (Hhf1/2), H2A (Hta1/2) and H2B (Htb1/2)—are <136 residues with isoelectric points >10.78. Canonical histone octamers are made up of two molecules of each histone, similar to septin octamers, but in a nonlinear arrangement (figure 4). The basic biochemistry of histone structure allows for strong electrostatic interaction with DNA and facilitates their function: to compact DNA into a highly condensed structure called chromatin that restricts accessibility to other molecules and cellular machinery. The complex including DNA wrapped around the octamer is called a nucleosome. Unlike the proteasome and septins, ‘incomplete’ (sub-octameric) histone assemblies are believed to have important functions during gene expression and the establishment of chromatin regions. For example, a histone hexamer with the H3–H4 tetramer and only one H2A–H2B dimer is thought to be an intermediate in nucleosome reassembly following transcription [49]. However, the specific assembly/disassembly pathways that yield these sub-octameric complexes remain unclear and widely unexplored. Here we focus on histone octamer assembly.

Histones individually expressed in *E. coli* (which lacks endogenous histones) aggregate into inclusion bodies, from which they can be isolated under denaturing conditions using affinity, size exclusion, and/or ion-exchange chromatography [50]. Removing denaturant allows the histones to refold, and if they are mixed together in equimolar amounts during refolding, the four core histones assemble spontaneously into 2:2:2:2 hetero-octamers. No chaperone or DNA is needed. High salt, however, is needed, with anions presumably neutralizing the positively charged residues that ultimately contact DNA and drive aggregation in the absence of DNA or high salt. Alternatively, co-expression of all four histones from a single polycistronic mRNA allows the assembly and purification (in high salt) of soluble octamers that are capable of interacting with DNA to produce nucleosomes [51].

What prevents histone aggregation during synthesis in the eukaryotic cytoplasm, where there is no DNA and the ionic strength is relatively low? ***General cytosolic chaperones*** bind co-translationally to H3, H2A and H2B in yeast translatome-wide selective ribosome profiling experiments [35]. As far as we can tell, co-translational assembly between distinct histone proteins has not been directly investigated. SEC and ion-exchange chromatography of cytosolic and nuclear fractions of human cells identified complexes containing H3 and general, ribosome-associated chaperones but no other histone [52]. Another cytosolic complex included H3, H4, a general chaperone of the Hsp90 family, and an Hsp90 co-chaperone [52]. This complex presumably represents an initial histone assembly step. (A series of post-translational modifications, beginning with co-translational acetylation, helps mark the timing of these assembly events relative to each other [52]; here we do not delve into these details.) The ‘final’ cytosolic complex includes the assembly chaperone Asf1 (anti-silencing function 1) [52], which binds to the H3–H4 dimer [48], and a karyopherin/importin protein that promotes subsequent nuclear import (figure 4).

As monomers, H2A and H2B are also bound co-translationally by ***general chaperones*** [35] followed by dimerization in the cytosol and interaction of the heterodimer with an ***assembly chaperone***, NAP1 (nucleosome assembly protein I) [53] (figure 4*a*). An importin then binds in a way that occludes H2A–H2B binding to nucleosomal DNA or to H3–H4 [54]. Importin also prevents non-specific non-nucleosomal interactions and therefore acts as a *bona fide* ***assembly chaperone***. Asf1 binding to H3–H4 heterodimers prevents the formation of H3–H4 tetramer by occluding the region on the histone H3 that is required for H3–H3 interaction in the context of an H3–H4 tetramer [48]. Hence as newly synthesized histone heterodimers enter the nucleus, they do not immediately assemble into octamers (figure 4).

At least during DNA replication and DNA repair, the CAF-1 histone ***assembly chaperone*** is thought to take over at this point: CAF-1 can receive H3–H4 dimers from Asf1 in a ‘hand-off’ mechanism that presumably involves binding between the two histone chaperones [55,56]. CAF-1 is sufficient to drive the assembly of (H3–H4)_2_ tetramers on DNA *in vitro* [57]. A recent X-ray crystallography study showed that in the absence of DNA, CAF-1 binds a single H3–H4 heterodimer in a way that should inhibit H3–H4 tetramerization [47]. When DNA is added, however, CAF-1 dimerizes in a manner that brings two H3–H4 dimers in proximity [47] (figure 4). Independent *in vitro* studies of yeast Nap1 showed that it binds with similar affinities to (H3–H4)_2_ tetramers as well as H2A–H2B dimers [58] but DNA easily outcompetes Nap1 for binding to (H3–H4)_2_ [59], supporting a model in which (H3–H4)_2_ tetramers deposit on DNA first, followed by incorporation of H2A–H2B dimers (figure 4). Cellular phenotypes upon CAF-1 depletion pointed to a requirement for DNA replication-coupled chromatin assembly [60], solidifying the model of DNA-driven assembly of histone octamer assembly along a stepwise pathway. Thus ***assembly chaperones*** and ***subcellular compartmentalization*** operate together to drive assembly order, according to the current model. This model was also created in part on the basis of isotope-labelling pulse-chase experiments done with cultured *Drosophila* cells and subsequent chromatin fractionation via sucrose gradients [61]. Further *in vitro* work using FRET, glycerol gradients and co-immunoprecipitation using purified histones, NAP1, CAF-1 and DNA [62,63] also supported the idea that interaction between H3–H4 tetramers and H2A–H2B dimers is the last step in histone octamer assembly [63].

As has been pointed out previously [53], there is limited direct evidence that this model of stepwise histone octamer assembly is generally applicable *in vivo*. One major complication is the semi-conservative nature of chromatin inheritance during DNA replication. Early isotope labelling experiments provided clear evidence that parental (H3–H4)_2_ tetramers stay together in the wake of the replication fork and octamers are replenished by the incorporation of two newly synthesized H2A–H2B dimers [64,65]. Transcription through a locus can also trigger incorporation of new histones, including non-canonical histone variants. Histone chaperones (as well as chromatin remodelers) are variously involved in both replication- and transcription-coupled histone octamer disassembly and reassembly, with most studies pointing clearly to replacement of parental H2A–H2B dimers with nascent H2A–H2B or variants thereof [66,67].

Does this mean that there is no physiological context for truly *de novo* histone octamer assembly? One scenario in which *de novo* assembly must occur is the replacement in the early zygote/embryo of protamines and other histone-like proteins that substitute for histones in compacting DNA into a transcriptionally silent manner in male gametes [68]. When the histone substitutes are destroyed prior to the initiation of zygotic transcription, the resulting naked DNA must be populated with histone octamers in a manner lacking any parental (H3–H4)_2_ ‘templates’. Indeed, the histone chaperone nucleoplasmin was first identified as a protein required for paternal chromatin decondensation [69]. Similarly, upon entry into cells, nucleosome-free viral DNA is rapidly bound by histones and assembled into nucleosomes independently of S phase [70,71]. Whether such *de novo* assembly takes place in the canonical order is unknown.

## 5. Predicting assembly order

The examples discussed above were chosen to highlight properties of multisubunit protein complexes that contribute to assembly order and, along the way, techniques that were used to establish assembly order. Box 1 summarizes commonly used experimental approaches to determine order. Other, less direct techniques have been used to predict assembly order for other complexes. The Path-LzerD method combines known structures of individual subunits with computational docking and calculation of predicted binding energies [72]. Its success was benchmarked against ‘known’ assembly pathways. However, the experimental methods that generated the ‘known’ pathways (e.g. contingencies during complex formation *in vitro* by full-length recombinant proteins refolded together following chemical denaturation [72,73]) are subject to many serious caveats. A non-computational approach relies on electrospray mass spectrometry (Box 1), which directly detects the mass and composition of subcomplexes [12,13]. However, assembly *per se* is not analysed; instead, pre-assembled complexes are observed during disassembly in ‘destabilizing’ buffer conditions, with the assumption that the assembly pathway is simply the reverse of the assembly pathway [13,74].

Studies based on this ‘((dis-)assembly)’ assumption concluded that interface area (the surface buried from solvent in an interface between subunits) drives assembly order: larger interfaces assemble first [13,74]. Thus order can be predicted by looking at interface size within the completed assembly. Using the ‘measure buriedarea’ command in ChimeraX [75], we calculated interface area for proteasome CP using cryoEM (PDB 8QYS [6]) and for septins using high-confidence computationally predicted structures (i.e. AlphaFold2 [76]). We found multiple incompatibilities with experimentally determined assembly order. For example, if we ask which individual proteasomal β subunit is predicted to add first to a pre-assembled ⍺ ring (which also includes the ‘proteasome maturation protein’, or POMP), interface area predicts β7 (area 1627 Å^2^). However, β7 is the final subunit to assemble. By far the largest yeast septin–septin interface in the octamer is Cdc3–Cdc12 (5875 Å^2^), yet according to CSD-BiFC, Cdc12 first binds Cdc11 (interface area 2237 Å^2^).

Likely reasons for the discrepancies come from the properties of multisubunit complexes that we have discussed here. First, the (dis-)assembly assumption likely fails for early steps in complexes that undergo co-translational assembly; consider the failure of full-length proteasome subunits Rpt1 and Rpt2 to properly interact, and the non-native oligomers formed by individually purified yeast septins (see above). Since much of the Cdc3–Cdc12 interface involves a coiled coil between the C-terminal sequences of both septins [27], if our co-translational model of septin assembly is accurate, the Cdc11–Cdc12 interface may fold before all of the sequences involved in the Cdc3–Cdc12 interface even exist. Second, chaperones are, by definition, not components of the final assembly, but they make major contributions to what interaction interfaces are available during assembly. Finally, the conformations of each subunit in the final assembly reveal few hints about the various other conformations they may have adopted during earlier assembly steps, which could have significant effects on the actual interfaces by which subunits interact during assembly (see figure 2B).

Caveats aside, predicting assembly order from final structures and relying on the (dis-)assembly assumption is clearly effective at revealing distinct mechanisms by which related complexes evolved [13,74]. A conceptually related evolutionary study exploited depletion of single subunits, with the idea that other subunits whose incorporation into the complex requires the missing subunit will be destabilized and destroyed [77]. Assembly order was investigated via the assumption that ‘subunits displaying stronger cooperative stabilization tend to form an intermediate subcomplex prior to further assembling into a larger complex because their respective monomeric states are labile’ [77]. As those authors acknowledged, there are challenges to verifying this assumption, but a fascinating outcome of the study was that paralogous complexes often evolve distinct assembly pathways [77]. Interestingly, in the few bacterial species that assemble a 20S proteasome CP (likely due to acquisition of genes via horizontal transfer from an archaeon) the assembly pathway seems to be distinctly different: rather than a self-assembling heptameric ⍺ ring templating subsequent addition of seven β subunits, the first assembly intermediate is an ⍺–β heterodimer, seven of which assemble via lateral interactions to form half of the final 28-subunit CP [78].

One way that assembly order can rapidly change during evolution is when two subunits become one: gene fusions can link together two distinct subunits into a single polypeptide [74]. Fusion presumably drives the two domains (formerly subunits) to associate first, since they are always in close physical proximity and are synthesized almost simultaneously. An intriguing example related to histones is the recent discovery that some viral genomes encode all four core histones linked together in a single polypeptide [79]. Such ‘quadruplets’ appear to be evolutionarily ancient; individual histones may have evolved later [79]. Using the viral linker sequences, the authors were able to link together eukaryotic histones, which allowed the linked histones to self-assemble during expression in *E. coli* into soluble ‘octamers’ capable of condensing DNA *in vivo* [79]. We note that the order of histones in the viral quadruplets is compelling: H3 and H4 are almost always adjacent, and the same is true of H2A and H2B [79]. Does order of translation in the linked polypeptide dictate order of ‘assembly’/association of the histone ‘subunits’/domains? Future research along these lines has the potential to provide new insights into assembly order for histones and beyond.

## 6. Final perspective

Perhaps due in part to the history of how multisubunit complexes were first studied, the default approach to thinking about how such complexes assemble focuses mostly on thermodynamics. In this way of thinking, subunits are building blocks with fixed shapes/geometries and they bind fastest/first to the other building blocks to which they stick the strongest. To extend this idea, the blocks are being shaken in a closed, otherwise empty box; they collide randomly with each other and the walls, and the non-productive collisions are instantaneous and have no effect on the blocks. In reality, assembly *in vivo* violates most of these assumptions. The building blocks are flexible and some are not even present when others start to interact. The box is also filled with a thick soup, the ingredients of which have non-negligible effects on the subunits when they encounter them. Some soup ingredients actually promote proper assembly by coating the building blocks in a way that prevents them from adopting irreversibly useless shapes or sticking to other soup ingredients in non-productive ways. Some blocks only interact in the right way when they are half-formed; if a block is tossed in the box after being manufactured elsewhere, it may never make the right interactions. Other blocks change their shapes periodically, with the period dependent on what ingredients are in the soup of the day (ask your waiter, and think GTPases and the GTP : GDP ratio). Still other blocks change their shapes upon interacting with another specific block. Finally, there is a mesh bag inside the box with pores that only some blocks can fit through. We hope this review raises awareness about how the properties of subunits can influence the assembly process and emphasizes the continued need for a more holistic understanding of how protein complex assembly works in living cells.

## Data Availability

This article has no additional data.
